# Targeting CGRP signaling alleviates cancer-associated pain in oral squamous cell carcinoma

**DOI:** 10.1186/s12903-026-08444-x

**Published:** 2026-04-29

**Authors:** Mingtao Chen, Yibo Guo, Zheqi Liu, Chengzhong Lin, Yang Wang, Xi Yang, Tong Ji, Chenping Zhang, Yu Zhang

**Affiliations:** 1 Department of Oral and Maxillofacial Surgery, Zhongshan Hospital, Fudan University, Shanghai, 200032 China; 2 Department of Oral Maxillofacial‑Head Neck Oncology, Shanghai Ninth People’s Hospital, Shanghai Jiao Tong University School of Medicine, Shanghai, 200011 China; 3 Department of Stomatology, Zhongshan Hospital, Fudan University, Shanghai, 200032 China

**Keywords:** Oral squamous cell carcinoma, Pain, Calcitonin gene-related peptide, Analgesic effect, Prognosis

## Abstract

**Background:**

Pain is the most frequent complication in patients with oral squamous cell carcinoma (OSCC) and is associated with poor prognosis. Calcitonin gene–related peptide (CGRP), abundantly expressed in sensory neurons, has been implicated in multiple pain disorders; however, its role in cancer-associated pain remains unclear. We investigated the relationship between CGRP and OSCC-associated pain and evaluated the analgesic potential of Rimegepant, a CGRP-receptor antagonist approved for migraine.

**Methods:**

Plasma CGRP was measured in OSCC patients and correlated with preoperative pain scores and clinical outcomes over a 30-month follow-up. Immunohistochemistry quantified CGRP within tumor-infiltrating nerve fibers. In a unilateral orthotopic tongue OSCC mouse model, we compared the analgesic effects of Rimegepant with carprofen and tramadol. RNA-sequencing of ipsilateral trigeminal ganglia from treated mice was performed to explore potential mechanisms.

**Results:**

Plasma CGRP levels were positively correlated with patient-reported pain severity and were associated with reduced overall survival. Increased CGRP expression in tumor-infiltrating nerves was linked to greater pain intensity and lymph node metastasis. In an OSCC mouse model, both systemic and local administration of Rimegepant significantly attenuated facial mechanical hypersensitivity. Transcriptomic analysis of trigeminal ganglia, together with RT–qPCR validation, revealed suppression of innate immune and neuroinflammatory pathways following Rimegepant treatment.

**Conclusions:**

Our findings link sensory-nerve-derived CGRP to OSCC-associated pain and support further evaluation of CGRP receptor antagonism as a potential analgesic strategy in cancer.

**Supplementary Information:**

The online version contains supplementary material available at 10.1186/s12903-026-08444-x.

## Introduction

Pain is one of the most prevalent concomitant symptoms observed in patients diagnosed with oral squamous cell carcinoma (OSCC). Given the extensive distribution of sensory nerves in the maxillofacial region, more than 40% of individuals with head and neck cancer experience early-stage pain, ranking it second in prevalence after pancreatic cancer [1]. OSCC-associated pain not only disrupts regular eating and sleeping, resulting in inadequate nutrition intake and physiological imbalances but is also an independent factor influencing the overall survival rate of patients [2, 3].

Currently, the World Health Organization (WHO) recommended a three-step analgesic ladder is the endorsed approach for managing cancer-associated pain. Nonetheless, a significant proportion of patients with cancer still do not receive reasonably effective pain relief, which can be partly attributed to the subjective preferences of physicians when selecting analgesic medications [4]. A global study indicated a 49% reduction in opioid doses prescribed by oncologists worldwide between 2010 and 2015 [5]. Moreover, patients in certain countries or regions, influenced by historical contexts and cultural factors, often possess a strong aversion to addiction and uphold a culture of enduring pain. Recent investigations have revealed that while opioids do provide pain relief, they also promote tumor development through mechanisms such as stimulating angiogenesis, facilitating cell cycle progression, migration and metastasis [6]. Considering the numerous challenges encountered in managing cancer-associated pain, there exists an urgent need for developing novel analgesic medications.

Increasing evidence suggests that neurons interact with cancer cells through paracrine or electrochemical communication in pan-cancer, regulating tumor progression and influencing drug resistance [7, 8]. We previously showed that cancer cells in low-glucose environments co-opt nociceptive nerves to drive production of calcitonin gene-related peptide (CGRP), and that this neurogenic CGRP induces cytoprotective autophagy in cancer cells thereby linking tumor metabolic stress to tumor–nerve crosstalk [9]. Moreover, subsequent work demonstrated that activation of tumor-innervating nociceptive neurons elevates CGRP secretion which remodels tumor-draining lymph nodes into an immune-suppressed state, connecting CGRP-driven neuronal signaling to tumor immune escape [10]. CGRP, which is highly expressed in C fibers and Aδ fibers of sensory nerves, not only regulates neural function directly but also exerts physiological effects by entering the bloodstream through neural paracrine secretion [11]. The plasma CGRP has been implicated in various pain-related diseases such as osteoarthritis, chronic pancreatitis, and migraine in previous studies [11, 12]. However, the role of CGRP in cancer-associated pain necessitates further exploration. Notably, a CGRP receptor antagonist named Rimegepant was recently approved by the FDA for the treatment and prevention of migraine, offering greater convenience for CGRP-related clinical and basic research [13, 14].

Here, using complementary clinical cohorts, histopathology, and a murine OSCC pain model, we examine the association between CGRP and cancer-associated pain and evaluate the analgesic and molecular effects of the CGRP-receptor antagonist Rimegepant, aiming to provide a theoretical foundation for CGRP as a prospective target for new analgesic drugs and contribute fresh perspectives on cancer pain management.

## Materials and methods

### Collection of OSCC patient samples

Two independent cohorts were enrolled at the Department of Oral and Maxillofacial Surgery–Head and Neck Oncology, Shanghai Ninth People’s Hospital. OSCC Group A (plasma cohort, *n =* 70) was collected between May 2020 and December 2020; OSCC Group B (tissue cohort, *n =* 79) was collected between June 2021 and January 2022. In both periods, patients with a histopathological diagnosis of OSCC were consecutively and randomly enrolled. Group A provided preoperative plasma and pain scores and was uniformly followed for 30 months post-surgery (primarily by scheduled outpatient visits, with telephone follow-up for missed visits); recurrence and metastasis were confirmed by imaging and, when available, pathology. Patients with uncontrolled hypertension, diabetes, or migraine were excluded to minimize confounding effects on peripheral CGRP. Group B provided formalin-fixed and paraffin-embedded tumor blocks for histopathology and nerve-focused histopathological and immunohistochemical analyses. Preoperative pain scores were assessed using the NRS within 1 day prior to surgery while patients were not using analgesics. Tumors were staged according to the AJCC/UICC 8th edition and graded per WHO criteria. Clinical data were retrieved from electronic medical records. Informed consent was obtained from all patients. The use of human specimens in this study was approved by the Institutional Research Ethics Committee of Shanghai Ninth People’s Hospital (No.SH9H-2019-T172-2).

### Enzyme-linked immunosorbent assay

Fresh venous blood samples from the subjects were collected in ethylenediaminetetraacetic acid tubes, stored at 4 °C, and processed within 2 h. The venous blood samples were centrifuged at 3000 rpm for 15 min to separate the plasma, which was then analyzed for human plasma CGRP content using human CGRP ELISA kits (E-EL-H0619c, Elabscience, China) according to the manufacturer’s instructions.

### Immunohistochemical analysis

Paraffin-embedded sections were subjected to deparaffinization, rehydration, antigen retrieval, endogenous peroxidase activity blocking, and nonspecific site blocking. Subsequently, the sections were individually incubated overnight at 4 °C with the following specific antibodies: rabbit monoclonal antibody against CGRP (1:100, #14,959, Cell Signaling Technology, USA) and mouse monoclonal antibody against PGP9.5 (1:1000, GB12159, Servicebio, China). Following thorough washing, goat anti-rabbit/mouse secondary antibodies labeled with horseradish peroxidase were applied and incubated at room temperature for 60 min. After washing, the sections were developed with diaminobenzidine and counterstained with hematoxylin. Finally, the sections were dehydrated and mounted.

For each patient, 3 non-overlapping sections were analyzed, and 5 randomly selected high-power fields (HPFs, 400 × magnification) were evaluated per section. All immunohistochemical staining results were independently reviewed by two experienced oral pathologists who were blinded to the clinical grouping.

Tumor-infiltrated nerve fibers were defined as previously described: (1) cancer cells invading nerve fibers; (2) tumor cells surrounding at least 33% of the nerve circumference; or (3) tumor cells infiltrating any layer of the nerve sheath structure [14].

CGRP-positive nerve fibers were identified based on distinct cytoplasmic brown staining within nerve structures that co-localized with PGP9.5-positive nerve fibers, with staining intensity clearly above background levels. Weak or ambiguous staining was excluded. The proportion of CGRP-positive nerves was calculated as the percentage of CGRP-positive nerve fibers among all tumor-infiltrated nerve fibers, allowing normalization across samples with variable tumor size and nerve density [15].

### Cell lines and culture

Human OSCC CAL27 and SCC25 cells were purchased from the American Type Culture Collection (USA). CAL27 cells were cultured in DMEM medium (Sourcelight, China), while SCC25 were cultured in a 1:1 mixture of DMEM and Ham's F-12 medium(Sourcelight, China). Both media were supplemented with 10% fetal bovine serum (Biological Industries, Israel) and 5% antibiotics (Neosyl, China). The cells were maintained in a cell culture incubator at 37 °C, 5% CO2, and high humidity.

### Unilateral tongue orthotopic transplantation model and application of analgesics

All animal studies were performed in accordance with the Guide for Care and Use of Laboratory Animals (The Ministry of Science and Technology of China, 2006) and the appropriate ethical regulations of Shanghai Ninth People’s Hospital. BALB/c nude mice were obtained from Jihui (Shanghai, China) and maintained under specific pathogen-free conditions. Both male and female mice were used in this study. All experimental procedures were approved by the Institutional Research Ethics Committee of Shanghai Ninth People’s Hospital (No.SH9H-2022-A896-1).

BALB/c nude mice aged 8 weeks were chosen to house in the SPF-level animal facility at Shanghai Jiao Tong University School of Stomatology, as BALB/c nude mice exhibit increased sensitivity to pain and a more stable tumor progression cycle, providing an advantage for intergroup comparisons. CAL27 and SCC25 cells were digested, centrifuged, and resuspended to a concentration of 2 × 10^6^ cells/ml. Mice were anesthetized with 1.25% tribromoethanol (0.02 ml/g, Nanjing Aibeibiotech, China) via intraperitoneal injection. A 25 μl cell suspension was submucosally injected into the left side of the tongue in 10 min. After inoculation, mice were maintained under standard conditions.

Starting from day 5 post-inoculation, mice were randomly divided into different groups and treated with analgesic drugs, including Carprofen (5 mg/kg), Tramadol (5 mg/kg) and Rimegepant (10 mg/kg or 50 mg/kg), via intraperitoneal injection once every 24 h. For local administration, Rimegepant was delivered via local lingual injection at a dose of 10 mg/kg. The dosages of Carprofen and Tramadol were determined based on Laboratory Animal Anaesthesia, while the dosage of Rimegepant(10 mg/kg) for mice was determined by converting the standard human dosage to mouse dosage based on body surface area. To prevent unnecessary pain to the mice and the risk of tumor invasion to the contralateral tongue, all mice were euthanized within 10 days after tumor inoculation. Euthanasia was performed under anesthesia (by intraperitoneal injection (20 mg/kg) of 1.25% tribromoethanol) using cervical dislocation method.

The administration of Carprofen and Tramadol was based on effective and safe dosages referenced from prior research. The dosage of Rimegepant (10 mg/kg) was calculated from human-recommended dosages converted to mice based on body surface area. Bilateral cheek mechanical sensitivities were measured before and 1 h after each treatment. The sample size was decided through pre-experiments. All injected solutions were freshly prepared on the day of the experiment. All mice were euthanized on the ninth day after tumor injection.

### Measurement of the mechanical threshold

Behavioral tests were conducted on BALB/c nude mice aged 8 weeks. To prepare the mice for mechanical sensitivity measurement, they were placed in restrainers and acclimated to the measurement environment for 30 min daily at room temperature (~ 22℃), from 1 week prior to the formal measurement. All behavioral assessments were performed by investigators blinded to group allocation, and group identities were concealed until completion of data analysis to minimize bias. During the formal measurement, mice were placed in restrainers, and the pistons of the restrainers were adjusted so that the cheek could protrude from the central opening of the piston, while still allowing the mouse to retract its head freely. After the mice calmed down and were no longer engaged in struggles or exploratory activities, repeated stimulation of the cheek was applied with a light force through a Von Frey filament to induce habituation to the stimulus behavior. Then, the 50% withdrawal threshold was determined using the up-down method of Dixon. In this paradigm, stimuli were always presented consecutively, whether ascending or descending. In the absence of a withdrawal response to the initially selected hair, a stronger stimulus was presented; in the event of withdrawal, the next weaker stimulus was chosen. The counting of the six critical data points did not begin until the response threshold was crossed. The 50% mechanical withdrawal threshold was calculated using the formula of Dixon [16, 17].

### Preparation of trigeminal ganglion samples

BALB/c nude mice (8 weeks old, weighing 20–25 g) were randomly divided into an experimental group and a control group (*n =* 3 in each group). Unilateral tongue orthotopic transplantation models were established for each group. In the experimental group, Rimegepant (50 mg/kg/day) was intraperitoneally injected every 24 h for 4 consecutive days, starting from the 5th day after tumor inoculation. The control group received an equal volume of physiological saline. After the final drug treatment, both groups of mice were euthanized, with the trigeminal ganglia on the side of the transplantation tumor carefully isolated. After rinsing with PBS, the trigeminal ganglia were preserved in a tissue storage solution.

### RNA extraction and RT-qPCR

Trigeminal ganglia were rapidly dissected, snap-frozen in liquid nitrogen, and stored at − 80 °C until RNA extraction. Total RNA was extracted from tissues using TRIzol reagent (TRIzol Reagent, Thermo Fisher Scientific) according to the manufacturer’s instructions. The concentration and purity of RNA were determined by spectrophotometry, and RNA integrity was assessed prior to downstream analysis. Complementary DNA (cDNA) was synthesized from 1 μg of total RNA using a reverse transcription kit (PrimeScript RT Reagent Kit, Takara Bio) following the manufacturer’s protocol. Quantitative PCR was performed using a SYBR Green Master Mix (TB Green Premix Ex Taq, Takara Bio) on a real-time PCR system (QuantStudio 5 Real-Time PCR System, Applied Biosystems). The amplification conditions were as follows: initial denaturation at 95 °C for 30 s, followed by 40 cycles of 95 °C for 5 s and 60 °C for 30 s. A melting curve analysis was performed to verify the specificity of amplification. Gene expression levels were normalized to an internal control gene (β-actin), and relative expression was calculated using the 2^−ΔΔCt^ method.

### RNA sequencing and analysis

Total RNA was extracted using TRIzol reagent (Invitrogen) according to the manufacturer’s protocols for the indicated samples. The cDNA libraries were then constructed for each pooled RNA sample using VAHTSTM Total RNA-seq (H/M/R). Differential gene and transcript expression levels from RNA sequences were examined using TopHat and Cufflinks. Gene expression levels were determined using the FPKM method. We applied the DESeq algorithm to screen for differentially expressed genes between the two groups. Moreover, GO enrichment and Kyoto Encyclopedia of Genes and Genomes (KEGG) analyses were performed to elucidate the biological functions of differentially expressed genes.

### Statistical analysis

All continuous variables were first examined for distributional assumptions using the Shapiro–Wilk test; homogeneity of variance was assessed where appropriate. Values are presented as mean ± standard error of the mean (SEM) for bar graphs and scatterplots. For box plots, the central line denotes the median, the box limits indicate the interquartile range (IQR), and whiskers represent the minimum and maximum values. For comparisons between two groups, normally distributed data were analyzed by an unpaired two-sided Student’s t-test; non-normally distributed data were analyzed using the Mann–Whitney U test. For comparisons involving more than two groups, one-way ANOVA was used for parametric data, and the Kruskal–Wallis test was used for nonparametric data. Two-way ANOVA (general linear model) was employed to evaluate main effects and interactions where two independent variables were present, followed by post-hoc testing as required. Overall and disease-free survival were calculated using the Kaplan–Meier method. Analyses were performed using GraphPad Prism 8 software (GraphPad Software Inc., USA). A two-sided P value < 0.05 was considered statistically significant. All data underwent the appropriate hypothesis tests.

## Results

### Elevated plasma CGRP levels correlate with OSCC-associated pain and poor clinical prognosis

**Fig. 1:**
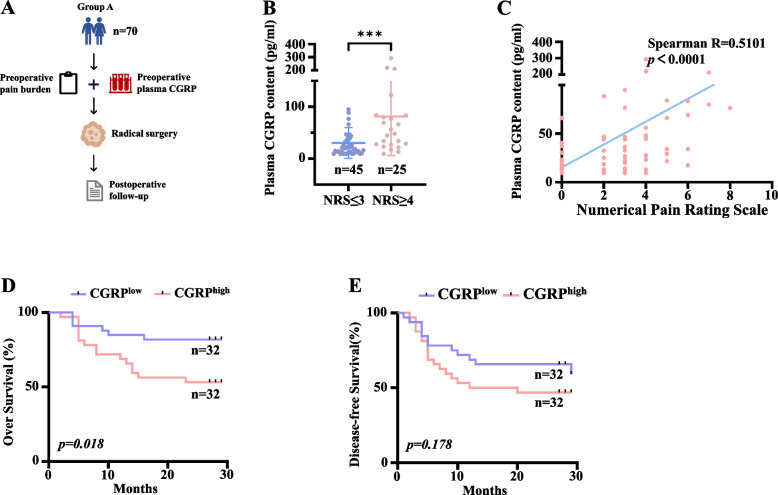
Elevated plasma calcitonin gene-related peptide (CGRP) levels were correlated with severe pain levels and poor prognosis in patients with oral squamous cell carcinoma (OSCC). **A** Study design of the OSCC-group A. **B** Plasma CGRP levels were compared between patients reporting no or mild pain (numeric rating scale (NRS) ≤ 3) and those experiencing moderate to severe pain (NRS ≥ 4), using unpaired two-tailed Student’s t test. ***P < 0.001. **C** The correlation between plasma CGRP levels and pain severity was analyzed using Spearman's rank correlation analysis (*n =* 70). **D**, **E** Kaplan–Meier survival analysis of overall survival rates and disease-free survival rates in patients with high and low plasma CGRP levels, stratified based on the median

In Group A (*n =* 70), patients reporting moderate-to-severe preoperative pain (NRS ≥ 4) had higher plasma CGRP than those reporting no or mild pain (NRS ≤ 3) (Fig. 1B; *P <* 0.001). Plasma CGRP correlated positively with preoperative pain severity (Spearman’s ρ; *P <* 0.001; Fig. 1C). After excluding four patients who did not undergo surgery and two who were lost to follow-up, the remaining 64 patients were dichotomized at the median CGRP concentration. Elevated plasma CGRP was associated with reduced overall survival (Fig. 1D; *P =* 0.018); a similar trend was observed for disease-free survival but did not reach statistical significance (Fig. 1E; *P =* 0.178). These findings underscore the associated between plasma CGRP levels, pain severity, and overall prognosis of patients with OSCC.

### CGRP expression in tumor-infiltrated nerves within OSCC tissue correlates with pain levels and adverse clinicopathological indicators

**Fig. 2:**
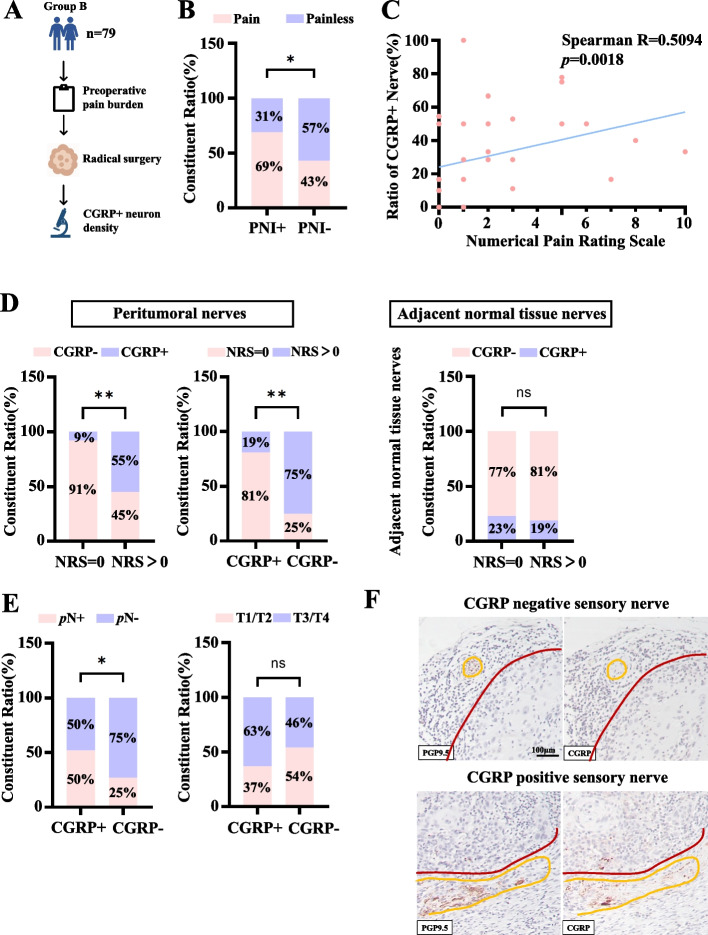
Analysis of CGRP expression in tumor-infiltrated nerves and its correlation with clinical pathological indicators. **A** Study design of the OSCC-group B. **B** The association between status of perineural invasion and OSCC-associated pain among 79 patients. **C** The correlation between the ratio of CGRP-positive nerves and pain severity was analyzed using Spearman's rank correlation analysis (*n =* 35). **D** The correlation between the pain status and expression of CGRP in peritumoral nerve fibers and adjacent normal tissue nerve fibers were analyzed using Fisher's exact probability test. **P < 0.01. **E** The correlation between lymph node metastasis and tumor staging with the expression of CGRP in tumor-infiltrated nerve fibers was analyzed using Fisher's exact probability test. **F** Representative IHC images of CGRP negative/positive nerve from OSCC-group B. *P < 0.05, ns *P >* 0.05. Scale bars: 100 μm. Nerve: Yellow, Tumor: Red

Tumor perineural invasion (PNI) is the pathological and physiological basis of nerve interaction in OSCC [18]. In Group B (*n =* 79) we first confirmed that patient-reported OSCC-associated pain was more frequent in tumors exhibiting PNI: 69% of PNI-positive cases reported preoperative pain versus 43% of PNI-negative cases (Fig. 2B). Because plasma CGRP is largely derived from neural paracrine secretion, we next quantified CGRP immunoreactivity within tumor-invading nerve fibers and examined its relationship with pain. Quantification of CGRP immunoreactivity within tumor-infiltrated nerves showed a positive association with pain severity (Spearman R = 0.5094, *P =* 0.0018; Fig. 2C). Consistent with this, patients whose tumor-invaded nerves were CGRP-positive were substantially more likely to report pain: 75% of cases with CGRP-positive nerves had NRS > 0 compared with 19% in the CGRP-negative group. In contrast, the proportion of CGRP-positive nerves did not differ significantly in the adjacent normal tissue (Fig. 2D). Moreover, CGRP positivity in tumor-infiltrated nerves was associated with a higher frequency of lymph node metastasis (*P <* 0.01), while no significant relationship was observed between nerve CGRP status and primary tumor T stage (*P >* 0.05; Fig. 2E). Representative serial sections stained for PGP9.5 and CGRP illustrate CGRP-negative and CGRP-positive sensory nerves adjacent to tumor (Fig. 2F). Together, these tissue-level findings align with our plasma analyses and support a link between neural CGRP expression and OSCC-associated pain.

### Rimegepant provides an effective and long-lasting analgesic effect on OSCC-associated pain

To clarify whether CGRP serves as a direct target for regulating OSCC-associated pain, we next conducted further validation using Rimegepant, the CGRP receptor antagonist with high specificity and minimal side effects, in animal models [19]. The modified Von Frey test was used to measure the mechanical thresholds of animals. Weber’s law shows that the ability to tell the difference between objects is proportional to the difference in their weights, which demonstrated that the Von Frey stimuli are evenly separated on a log-gram scale. To gain a more intuitive understanding of the changes in mechanical sensitivity in mice, we calculated the differences in logarithm of mechanical threshold values before and after drug administration and defined them as “changes in sensitivity”, thereby reflecting the change in mechanical sensitivity.

**Fig. 3:**
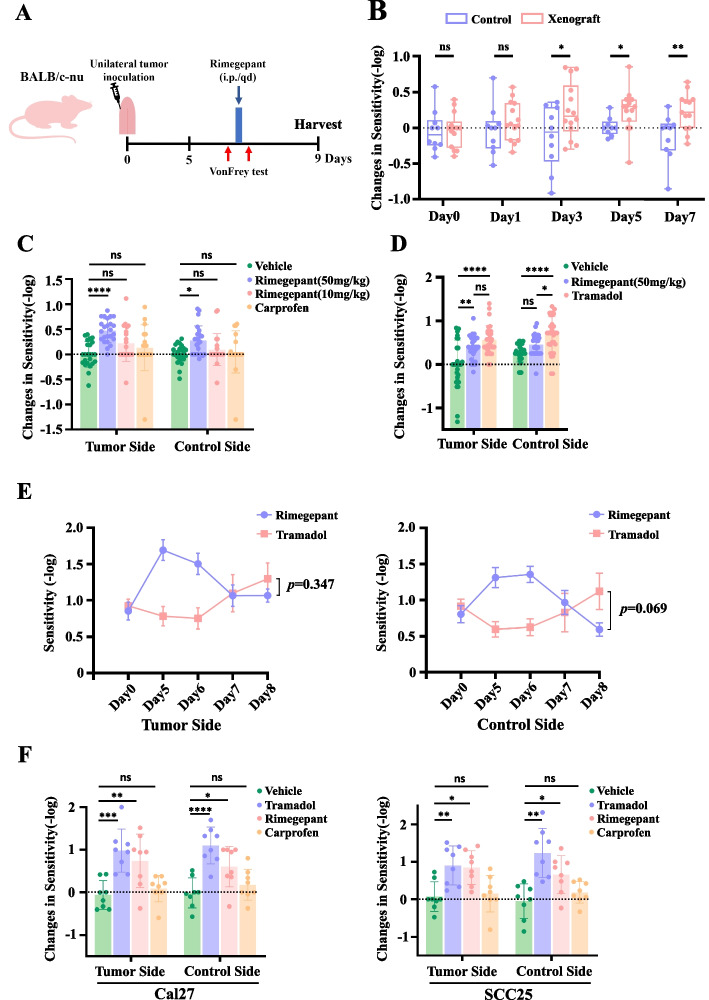
Analgesic effects of Rimegepant in OSCC-associated pain demonstrated via the unilateral tongue orthotopic transplantation model. **A** Construction of the unilateral tongue orthotopic transplantation model and drug injection with mechanical threshold measurement protocol. **B** Following tumor transplantation, the differences in mechanical sensitivity between the transplanted (*n =* 14) and non-transplanted (*n =* 10) sides were compared using unpaired two-tailed Student’s t test. **C** Changes in sensitivity 1 h after Rimegepant (50 mg/kg), Rimegepant (10 mg/kg) and Carprofen treatment were performed using one-way ANOVA. Each dataset included results from monitoring over 4 consecutive days (*n =* 6–8/group). **D** Changes in sensitivity following Rimegepant (50 mg/kg) and Tramadol treatment at 1 h were performed using one-way ANOVA. Each dataset included results from monitoring over 4 consecutive days (*n =* 6–8/group). **E** The 4-day trends of facial mechanical sensitivities 24 h after Rimegepant (50 mg/kg) and Tramadol treatment. (*n =* 6–8/group). **F** Changes in sensitivity 1 h after Tramadol, Carprofen treatment and local lingual injection of Rimegepant (10 mg/kg) in CAL27 and SCC25 xenograft models were performed using one-way ANOVA(*n =* 8). *P < 0.05, **P < 0.01, ***P < 0.001, ****P < 0.0001, ns *P >* 0.05

Initially, we established a murine model of unilateral tongue orthotopic transplantation tumors in BALB/c nude mice and subsequently validated its accuracy using the modified Von Frey test (Fig. 3A), which avoided visual interference with the withdrawal response when stimulation of the cheek was applied.

The outcomes revealed a noteworthy increase in facial mechanical sensitivity on the tumor-affected side compared to the control side (Fig. 3B), corroborating that unilateral tongue orthotopic transplantation tumor induced mechanical sensitization in the ipsilateral facial region of mice. The mechanical sensitization effect became evident after the third day. These results indicate the successful construction of the unilateral facial cancer pain model.

Based on the model, we compared the analgesic effects of Rimegepant against Carprofen, the representative NSAID, and Tramadol, the representative weak opioid drug. The results indicated that neither Rimegepant (10 mg/kg) nor Carprofen provided significant relief for OSCC-associated pain (Fig. 3C). However, Rimegepant at a dose of 50 mg/kg demonstrated a notable reduction in facial mechanical sensitivity 1 h after treatment, although its efficacy was inferior to that of Tramadol (Fig. 3D). And the results of one-way ANOVA indicated that this effect of Rimegepant (50 mg/kg) was more pronounced on the tumor-affected side compared to the control side. We then compared the mechanical sensitivity changes after 24 h of treatment with Rimegepant (50 mg/kg), considering its 11-h half-life and 96% plasma protein binding rate in humans [20]. The results showed Rimegepant appeared to exhibit a more sustained analgesic effect compared with Tramadol, although no statistically significant difference was observed. (Fig. 3E). To further exclude potential systemic effects, we assessed the analgesic efficacy of locally administered Rimegepant. Consistent results were observed in both CAL27 and SCC25 xenograft models, where low-dose(10 mg/kg) local administration of Rimegepant effectively alleviated cancer-associated pain.

### Rimegepant modulates OSCC-associated pain by altering the innate immune status of the trigeminal ganglion

**Fig. 4:**
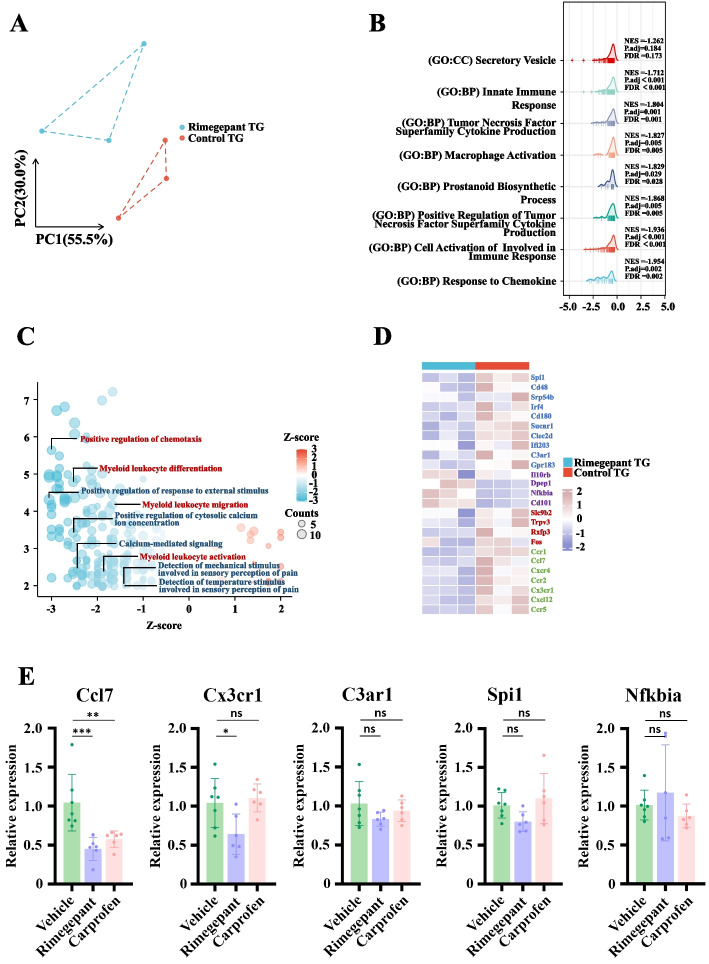
RNA sequencing and analysis of the trigeminal ganglia in tumor-bearing mice treated with Rimegepant. **A** Principal component analysis of transcription levels in trigeminal ganglia following Rimegepant treatment. **B** Gene set enrichment analysis (GSEA) screened out items downregulated in the trigeminal ganglia following Rimegepant treatment. **C** GO-KEGG analysis screened out items downregulated in the trigeminal ganglia following Rimegepant treatment (logFC > 2 or < 0.5, *P <* 0.05). **D** Transcription levels of indicated genes in the trigeminal ganglia following Rimegepant treatment. Genes with different functions are represented by different colors: pro-inflammatory genes in blue, anti-inflammatory genes in purple, neuroactive genes in red, and chemokine genes in green. **E** Relatively expressed genes in the trigeminal ganglia of BALB/c nude mice after different treatments. *P < 0.05, **P < 0.01, ***P < 0.001, ns *P >* 0.05

Although the role of CGRP in OSCC-associated pain is well established, the specific mechanism by which Rimegepant reduces OSCC-associated pain remains unclear. The trigeminal nerve is primarily responsible for facial sensation and exhibits the highest expression of CGRP. Therefore, we explored the impact of Rimegepant on the transcriptome of the trigeminal ganglion in mice and found that the transcription profiles changed significantly following treatment compared to the control group (Fig. 4A). Following Rimegepant administration, we observed a significant downregulation of innate immune-related items within the trigeminal ganglia, as well as functional items directly associated with pain perception and calcium signaling pathways (Fig. 4B, C). The activation of innate immune within the trigeminal ganglia is known to enhance nociceptive responses, which were also directly linked to the activation of calcium signaling pathways [20–22]. These results suggest that Rimegepant alleviates OSCC-associated pain by mitigating the inflammatory condition within the trigeminal ganglion.

Finally, we categorized the genes enriched in these functional groups. Genes that promote the proliferation and differentiation of innate immune cells, and those related to the expression of proinflammatory mediators, were significantly downregulated, while those associated with anti-inflammatory functions were significantly upregulated. Additionally, genes from various chemokine receptor families and ion channel genes linked to neuronal activity were significantly downregulated (Fig. 4D). To validate these findings, RT–qPCR was performed to assess the expression of innate immune-related genes in the trigeminal ganglia of BALB/c nude mice following different analgesic treatments. Notably, Rimegepant treatment led to a reduction in the expression of Ccl7 and Cx3cr1. These results further support that Rimegepant may alleviate cancer-associated pain, at least in part, by suppressing innate immune activation within the trigeminal ganglia. Collectively, our findings suggest that modulation of neuro-immune interactions represents a potential mechanism underlying the sustained analgesic effect of Rimegepant.

## Discussion

Although the pivotal role of CGRP in numerous pain-related conditions has been well established [21, 22], its role in cancer-associated pain remains ambiguous. In this study, we identified CGRP as a significant neuropeptide involved in the modulation of OSCC-associated pain. We also demonstrated the potential of the CGRP receptor antagonist, Rimegepant, as a novel analgesic agent that warrants further development. Initially, we established the relationship between plasma CGRP levels and OSCC-associated pain through clinical investigations. In our cohort, plasma CGRP levels were positively correlated with pain severity and were associated with poorer overall survival. Notably, in our previous independent clinical cohort, plasma CGRP levels were significantly higher in OSCC patients than in healthy controls (48.59 vs. 14.58 pg/ml, *P <* 0.0001) [23], further supporting the disease-associated elevation of circulating CGRP. Together, these findings reinforce the potential role of CGRP as both a biomarker of cancer-associated pain and a prognostic indicator in OSCC. Previous research has recognized plasma CGRP as a notable biomarker linked to diverse pathological and physiological states, including blood pressure regulation, atherosclerosis, and arthritis [11]. Some researchers have noted elevated plasma CGRP levels in conditions characterized by pain, such as trigeminal neuralgia, osteoarthritis, and chronic pancreatitis. Our research contributes to the body of evidence suggesting that plasma CGRP could serve as a pivotal biomarker of the status of cancer-associated pain in patients with OSCC. Additionally, numerous studies have reported heightened plasma CGRP levels in patients with breast, lung, and prostate cancer [24–26], which have been linked to poor prognosis. Similarly, we found that plasma CGRP also exhibits potential as a prognostic predictor in OSCC through clinical data.

The neurogenic release of CGRP, facilitated by paracrine mechanisms, represents the primary origin of plasma CGRP. Coincidentally, perineural invasion represents a prevalent pathological characteristic in OSCC, with previous studies demonstrating that neural infiltration is associated with OSCC-associated pain. Nagamine et al. [27] observed upregulated expression of CGRP within the trigeminal nerve in an orthotopic OSCC transplantation tumor model, concomitant with facial mechanical sensitization. Likewise, Bloom et al. identified a correlation between the presence of CGRP-positive sensory nerves and metastatic bone pain in a breast cancer bone metastasis model [28]. Consequently, further investigation is required to elucidate the relationship between cancer-associated pain and CGRP expression within infiltrating nerve fibers. Our immunohistochemical staining results demonstrated a correlation between preoperative pain level and risk of lymph node metastasis with CGRP expression in tumor-infiltrated nerve fibers in patients diagnosed with PNI. These findings provide crucial histopathological evidence supporting the involvement of CGRP in cancer-associated pain.

The availability of CGRP receptor antagonists on the market has significantly facilitated our research efforts. Here, we validated the analgesic properties of the CGRP receptor antagonist, Rimegepant, in mouse models of OSCC-associated pain. It is important to note that while we observed significant analgesic effects in mice using a Rimegepant dose that was five times higher than the standard human dose, Mallee et al. [29] demonstrated that due to structural differences in receptor activity-modifying protein 1 within CGRP receptors, the affinity of non-peptide CGRP receptor antagonists for human CGRP receptors is 100 times greater than that for rodent receptors. This indicates that Rimegepant has the potential to provide and maintain effective analgesia in humans at safe dosages.

Based on the current consensus in pathophysiology, pain can be classified into two main categories: inflammatory pain, which results from the activation of nociceptive receptors due to tissue damage, and neuropathic pain, which arises from injuries or direct damage to the sensory nervous system [30, 31]. The mechanisms underlying the analgesic and migraine-preventing effects of CGRP receptor antagonists through the trigeminal-vascular system have been clarified. Nevertheless, given the complexity of cancer pain mechanisms, further exploration is warranted to comprehend the potential influence of CGRP receptor antagonists on cancer-associated pain. Studies have reported that the expression of pro-inflammatory factors induced by trigeminal ganglion injury is linked to ectopic pain in the orofacial region [32, 33]. CGRP can stimulate the expression of genes related to pro-inflammatory factors within the trigeminal ganglia and neuroglial cells, leading to heightened activity in nociceptive sensory neurons, as well as star-shaped glial cells and small glial cells in the spinal dorsal horn [34, 35]. Our RNA-seq findings further support this notion, indicating that Rimegepant attenuates innate immune responses in the trigeminal ganglion while downregulating genes associated with calcium channels, thereby potentially reducing neuronal excitability. Given that CGRP receptors are predominantly expressed in glial cells and a specific subset of medium-sized neurons within the trigeminal ganglion, we propose that CGRP contributes to OSCC-associated pain by modulating neuro-immune interactions, particularly through regulating innate immune responses in both trigeminal neurons and glial cells. However, further experimental validation of this potential mechanism is warranted.

## Conclusion

In summary, our study demonstrates that circulating CGRP is closely associated with pain severity and clinical prognosis in patients with OSCC, and that CGRP expression in tumor-infiltrating nerves correlates with cancer-associated pain. Functionally, pharmacological blockade of CGRP signaling with Rimegepant effectively alleviated cancer pain in preclinical models, with evidence suggesting involvement of neuro–immune modulation within the trigeminal ganglion. Collectively, these findings identify CGRP as a key mediator of OSCC-associated pain and support the therapeutic potential of CGRP receptor antagonists as a novel strategy for cancer pain management.

### Limitations of the study

This study has several limitations. First, the use of BALB/c nude mice limits the interpretation of neuro–immune interactions, which should be further validated in immunocompetent models. Second, clinical data were constrained by the lack of non-cancer pain controls and limited longitudinal follow-up samples. Third, although validation was performed in two OSCC cell lines, broader confirmation across additional models is warranted. Finally, while tramadol did not show overt sedative effects during testing and prior studies suggest minimal motor impairment at analgesic doses, we did not perform dedicated behavioral assays (e.g., rotarod), and central effects cannot be completely excluded.

## Supplementary Information

Supplementary Material 1.

Supplementary Material 2.

Supplementary Material 3.

## Data Availability

The RNA-sequencing datasets analysed during the current study are available in the NCBI Gene Expression Omnibus (GEO) repository under accession number GSE260740 (secure token: kjwlagkkvpunfkt). Other datasets generated and/or analysed during the current study are not publicly available due to potentially identifiable/sensitive patient information, but are available from the corresponding author on reasonable request and following institutional review board approval and a data use agreement.
