# Supplementary material for: Targeting CGRP signaling alleviates cancer-associated pain in oral squamous cell carcinoma
Source: BMC Oral Health. 2026 Apr 29;26:857. doi: 10.1186/s12903-026-08444-x (PMC13173713; doi:10.1186/s12903-026-08444-x)
Supplement: Supplementary file 1 — Supplementary Material 1. [file 12903_2026_8444_MOESM1_ESM.docx]

Table S1. Demographics of patients in Group A

| Variable | No.(n=70) |
| --- | --- |
| Sex: |  |
| male | 50 |
| female | 20 |
| Average age | 59.2±14.0 |
| Tumor location: |  |
| Tongue | 29 |
| Cheek | 16 |
| Gingiva | 12 |
| Palate | 1 |
| Mouth floor | 6 |
| Mandible and maxilla | 6 |
| T stage: |  |
| T1/T2 | 33 |
| T3/T4 | 37 |
| N stage: |  |
| pN0 | 36 |
| pN+ | 34 |
| History of malignancy: |  |
| Primary | 62 |
| Recurrent | 8 |
| Perineural invasion: |  |
| PNI(-) | 43 |
| PNI(+) | 27 |

^a^ CGRP: calcitonin gene-related peptide

^b^ PNI: perineural invasion
