# Supplementary material for: Targeting CGRP signaling alleviates cancer-associated pain in oral squamous cell carcinoma
Source: BMC Oral Health. 2026 Apr 29;26:857. doi: 10.1186/s12903-026-08444-x (PMC13173713; doi:10.1186/s12903-026-08444-x)
Supplement: Supplementary file 2 — Supplementary Material 2. [file 12903_2026_8444_MOESM2_ESM.docx]

Table S2. Demographics of patients in Group B

| Variable | No.(n=79) |
| --- | --- |
| Sex: |  |
| male | 49 |
| female | 36 |
| Average age | 65.0±12.2 |
| Tumor location: |  |
| Tongue | 30 |
| Cheek | 10 |
| Gingiva | 20 |
| Palate | 4 |
| Mouth floor | 7 |
| Mandible and maxilla | 3 |
| Lip | 5 |
| T stage: |  |
| T1/T2 | 38 |
| T3/T4 | 41 |
| N stage: |  |
| pN0 | 51 |
| pN+ | 28 |
| History of malignancy: |  |
| Primary | 54 |
| Recurrent | 25 |
| Perineural invasion: |  |
| PNI(-) | 44 |
| PNI(+) | 35 |

^a^ PNI: perineural invasion
